# Prevalence of Dangerousness to the Public, Criminogenic Risk Factors and Psychopathic Traits in Child-to-Parent Offenders and Contrast with Non-Child-to-Parent Offenders

**DOI:** 10.3390/healthcare12060622

**Published:** 2024-03-09

**Authors:** Ricardo Fandiño, Juan Basanta, Francisca Fariña, Ramón Arce

**Affiliations:** 1 Department of Developmental and Educational Psychology, University of Santiago de Compostela, 15872 Santiago de Compostela, Spain; ricardo.fandino@usc.es; 2 Juvenile Court of Ourense, 32003 Ourense, Spain; basanta@cop.es; 3 AIPSE Departament, Universidade de Vigo, 36310 Vigo, Spain; francisca@uvigo.es; 4 Department of Political Science and Sociology, Faculty of Psychology, University of Santiago de Compostela, 15872 Santiago de Compostela, Spain; 5 Unidad de Psicología Forense, Faculty of Psychology, University of Santiago de Compostela, 15872 Santiago de Compostela, Spain

**Keywords:** criminogenic needs, juvenile offenders, intervention programs, recidivism prevention, criminal recidivism

## Abstract

Aim: Child-to-parent offenders (CPOs) are commonly specialist offenders and with high rates of recidivism. Thus, a field study was designed to estimate the prevalence of recidivism in the reference measures of recidivism i.e., dangerousness, risk factors and psychopathy, and compare CPOs with non-child-to-parent juvenile offenders (non-CPOs). Method: A total of 136 juvenile offenders (76.5% boys), 76 CPOs and 60 non-CPOs, aged from 14 to 18 years old, were measured in terms of dangerousness, risk factors and psychopathic traits. Results: For CPOs, the results show a more than common prevalence (>0.50), 75.0%, 95% CI [0.653, 0.847]) of dangerousness (caseness); a significant prevalence (>0.05) of diagnostic psychopathy (25.0%, 95% CI [0.150, 0.350]); and a common prevalence (=0.50), 55.3%, 95% CI [0.441, 0.665]) of classifications of high- and very high-risk factors. Comparatively, no significant differences were observed between CPOs and non-CPOs in terms of mental health problems (dangerousness); meanwhile CPOs exhibited significantly more interpersonal and affective psychopathic traits and significantly higher risks in family circumstances/parenting, and personality and behavior risk factors. Conclusions: The implications for prevention and intervention programs with CPOs are discussed.

## 1. Introduction

Three constructs have been applied to predict recidivism in violent criminal behavior: dangerousness, risk factors and psychopathy. Dangerousness, which has neither a shared denomination (e.g., dangerousness as a seminal and broad term, dangerousness to self or others, dangerousness to others, dangerousness to the public) nor a consistent definition (different legislations define it in different ways) over time in the targeted behavior (ranging from fuzzy “harmful acts” to “dangerous to others”), is referred to as the stable predisposition sustained in constitutional, personal internal factors (severe personality disorder) to commit criminal actions. This is a clinical issue demanded to mental health professionals when classifying individuals―dangerous or not dangerous―as prone to commit dangerous actions. In consequence, this predictive model is valid only for mental illness offenders. Nevertheless, effective interventions with offenders are mainly focused on training offenders in criminogenic needs [1,2,3], not in severe clinical disorder treatment (non-criminogenic need). Moreover, dangerousness as a personal attribute has shown a limited validity for the prediction of harmful acts [4,5]. The psychometric gold standard used to measure severe personality disorders in a forensic setting is the MMPI [6,7,8].

The construct of risk factors was originally proposed for forecasting the recidivism of property offenders themselves and later extended to other crimes. These consist in forecasting delictive behaviors based mainly on the identification of the risk factors, both contextual and personal factors, empirically observed as predictors [1,9,10,11]. The most powerful predictors of the risk of recidivism are family circumstances, substance abuse and personality behavior [12,13]. The literature’s reference for measuring risk factors is the Youth Level of Service/Case Management Inventory [14,15].

The construct of psychopathic traits (interpersonal, affective, lifestyle and antisocial traits) has also been associated with severe psychopathology, although the relationship between criminal behavior and severe psychopathology that has been proposed is not causal and, once present, is stable [16,17]. In any case, psychopathic offenders are at high risk of reoffending [18,19,20]. The Psychopathic Checklist-Revised (PCL-R; [21,22]), although it was not originally designed to measure the risk of recidivism, is the gold standard for measuring these traits [23].

The observed prevalence of child-to-parent violence ranges from occasional prevalence (criterion for occasional adverse effects: <0.05) to close to a normal prevalence (0.95). In other words, the victimization of child-to-parent violence spans from occasional actions affecting less than 5% of the population to normal actions involving close to 95% of the population [24]. Thus, the range of the estimations of the prevalence of child-to-parent violence is so large that estimations are explained by moderators. The most powerful moderators identified by the literature review were the definition of child-to-parent violence (i.e., physical, psychological, emotional), the frequency of the violent actions (one occasion ―zero tolerance criterion) vs. reiterated (chronic) and the target of the violent actions (father vs. mother). In relation to the definition of the violence, the highest rates were registered for psychological (e.g., 61.3% [25]), verbal (shouting, insults and threats, e.g., 53.5 for boys and 57.5% for girls [26]) and emotional (e.g., 46% [27]); the lowest, for physical (e.g., 5% [25]); and a medium rate in economic violence (e.g., 14.1% [25]). Other relevant moderators studied were the gender of the victim with higher rates for mothers than fathers, and the gender of the perpetrator with higher rates for boys than girls. However, these appraisals are tentative due to measurement errors. Thus, some studies on physical violence were actually measuring physical and psychological (i.e., threats) violence; another, technical violence (the responses to the frequency to any item were “sometimes” or further); meanwhile, another, the zero tolerance (a response different from “never” for any item). Hence, different rates of prevalence have been registered for each measure of physical violence. As for psychological violence, the literature has been using the same construct to measure psychological violence, verbal violence, and emotional violence [28]. These three types of violence are psychological violence, as the resulting harm for all of them is psychological [29]. Amazing rates (±95%, i.e., child-to-parent violence is normal in the population) for the prevalence of psychological violence with zero tolerance have been reported [24]. For psychological violence, the application of the zero-tolerance criterion is problematic, as a periodicity (reiteration) of actions is required to produce victimization [30], and without victimization, there is no victim and, hence, psychological violence [29]. In any case, infrequent (≤0.01) prevalence rates have not been reported. Contrariwise, there is no doubt that the prevalence of child-to-parent violence is statistically significant and a criminological problem. And about 1/3 of CPOs reoffend [31,32,33].

Keeping in mind that child-to-parent violence is a criminological problem and its association with recidivism, a field study was designed to estimate the prevalence of dangerousness, psychopathic traits and risk factors in child-to-parent offenders (CPOs). In addition, a comparison between CPOs and non-CPOs in terms of dangerousness, psychopathic traits and risk factors was performed.

## 2. Materials and Methods

### 2.1. Participants

Participants were 136 juvenile offenders (see in Table 1 the distribution of disposition orders), aged from 14 to 18 years old (M = 16.42, SD = 0.95), 76 sentenced by child-to-parent offenses and with no records for other crimes, 67.1% (*n* = 51) boys and 30.3% (*n* = 23) recidivist, and 60 for other crimes (non-CPOs), 88.3% boys, and 50.0% recidivist. Table 2 summarizes the distribution by crimes in non-CPOs.

### 2.2. Procedure and Design

Participants’ evaluation was court mandated to design the intervention with juvenile offenders. Two clinical psychologists with more than 10 years of experience in the Youth Offending Teams (YOTs) carried out the assessment protocols with participants (self-reports), with their families (interviews), with the judicial and institutional files, and with the social services. Participants were evaluated individually and face to face (self-reports and interviews) while they were serving their sentences. The order of the administration of the measurement instruments was rotated (standard rotation procedure) between participants to counterbalance a potential interaction effect among measures. Data were provided by Galician (northwest of Spain) courts (judicial files) and Young Offender Institutions.

The sensitivity of the design for a MANOVA with 2 groups and 8/4 response variables and a sample of 136 participants, the probability of detecting (1–β) significant differences (α < 0.05) of a moderate effect size (η^2^ = 0.059) is 99.9% and 99.3%, respectively. Likewise, the sensitivity of the contrast of an observed proportion with a constant (0.05 and 0.5) for a sample size of 76 participants, the probability of detecting significant differences of a moderate effect size (*OR* = 2.47/*g* = 0.212) is 95.8% and 99.9%, respectively.

### 2.3. Measure Instruments

Dangerousness to public, assessed as clinical syndromes, was measured with the basic clinical scales of the Spanish adaptation of the MMPI-A [34]. The MMPI-A basic clinical scales consist of 10 measures. The Masculinity-Femininity and the Social Introversion scales were disregarded because they do not measure clinical syndromes. The remaining 8 clinical scales measure: Hypochondriasis (Hs), Depression (D), Hysteria (Hy), Psychopathic deviate (Pd), Paranoia (Pa), Psychasthenia (Pt), Schizophrenia (Sc) and Hypomania (Ma). Complementary, as invalid protocols must be suspected in these populations acquiescence, lack of collaboration or outliers, the ? (Cannot Say), TRIN (True Response Inconsistency), VRIN (Variable Response Inconsistency), F, F1, F2, L and K scales were scored. The reported reliability, test-retest and internal consistency, was, for males and females respectively, 0.79 and 0.79 for Hypochondriasis, 0.78 and 0.66 for Depression, 0.70 and 0.59 for Hysteria, 0.80 and 0.66 for Psychopathic Deviate, 0.65 and 0.58 for Paranoia, 0.83 and 0.85 for Psychasthenia, 0.83 and 0.89 for Schizophrenia, and 0.70 and 0.61 for Hypomania. Construct validity was also reported.

Psychopathic traits were assessed with the Spanish adaptation [35] of the Hare Psychopathy Checklist: Youth Version (PCL:YV; [36]) which is, in turn, an adaptation to youngers of the adult version of the Psychopathy Checklist-Revised (PCL-R; [21]). The checklist consists of 20 items scored by clinicians (clinical judgments) on a 3-point Likert scale (from 0 = Does not apply at all, 1 = Partially applies, to 2 = Definitely applies) designed to assess psychopathic traits in adolescents (the Spanish adaptation ranges from 14 to 24 years). The PCL:YV is structured in four dimensions: interpersonal traits (i.e., superficial, grandiose, manipulative), affective traits (i.e., callousness, lacking remorse), behavioral traits (i.e., impulsivity, lacking goals), and antisocial traits (i.e., criminal versatility and serious criminal behavior). The Spanish adaptation of this checklist has showed an excellent reliability (ω = 0.94), and construct (for three and four-factors), convergent and discriminant validity. Likewise, inter-rater agreement has been reported as excellent for the total score: single rating ICC = 0.71 to 0.90 and averaged ratings ICC = 0.83 to 0.95. For the diagnosis of psychopathy (the observed reliability 0.94 validated the measure for applied settings where important decisions are making as diagnosis [37]), a cutoff score of 30, a conservative criterion of the PCL-R, was assumed [38] given that there is no a specific criterion for PCL:YV.

Criminogenic risk factors were evaluated with the Spanish adaptation [39] of the Youth Level of Service/Case Management Inventory (YLS/CMI; [14]. The YLS/CMI comprises 42 items which measure 8 criminogenic risk domains: (1) Prior Offenses and Dispositions (prior dispositions, prior probation, prior custody, admissions, probation violation, escape from custody, failure to appear); (2) Family Circumstances and Parenting (inadequate supervision, difficulty controlling behavior, inappropriate discipline; inconsistent parenting, father/mother poor relations); (3) Education and Employment (disruptive classroom behavior, disruptive schoolyard behavior, low achievement, problems with peers, problems with teachers, truancy, unemployed, not seeking employment); (4) Peer Relationships (casual friends are offenders or exhibit antisocial attitudes, close friends are offenders or exhibit antisocial attitudes, no/few positive acquaintances, no/few positive friends); (5) Substance Abuse (occasional drug use, chronic drug use, chronic alcohol use, substance abuse interferes with life, substance use linked to offense); (6) Leisure and Recreation (limited organized activities, waste time, no personal interests); (7) Personality and Behavior (inflated self-esteem, physically aggressive, tantrums, short attention span, poor frustration tolerance, inadequate guilt feelings, verbally aggressive); (8) Attitudes and Orientation (antisocial/pro-criminal attitudes, not seeking help, actively rejecting help, defies authority, little concern for others). Items are scored dichotomously (0 or 1), ranging the total score from 0 to 42. The reliability for the total score was excellent, α = 0.92, and evidence of construct validity was also reported.

### 2.4. Data Analysis

The inter-rater (two clinician evaluated separately the juvenile offenders in the YLS/CMI and the PCL:YV) reliability was assessed with Intraclass Correlation Coefficient (ICC). The levels of inter-rater reliability values were interpreted as poor (<0.50), moderate (0.50–0.75), good (0.75–0.90) and excellent (>0.90) [40], and the coefficient as the percentage of the true mean and variance.

A MANOVA was performed for the mean comparison in dangerousness ($\bar{r}$ = 0.514), psychopathic traits ($\bar{r}$ = 0.218) and criminogenic risk factors ($\bar{r}$ = 0.283) by the factor population (CPOs vs. non-CPOs). Effects size for multivariate effects was estimated with partial eta squared $(\eta_{p}^{2}$) and for univariate effects with *d* (Hedges’ unbiased formula), interpreting the magnitude according to Cohen’s [41] criteria. The statistical model error (i.e., the probability of misclassifications derived from with the application of the resulting model) was appraised with the Probability of an Inferiority Score (PIS; [42]). A derivation of the BESD [43] was used to quantify the incremental (decremental for negative values) effect in univariate effects in CPOs over non-CPOs [44].

The prevalence of caseness in clinical disorders was quantified in relation to clinical significance criterion (T score ≥ 66.45) and the observed probability was contrasted (Z) with a constant (trivial effect: 0.05; common effect: 0.50; [45]) to estimate the significance of the prevalence in the population of CPOs. Effect size was estimated with Odds Ratio (OR), being interpreted as small = 1.44, effect higher than 55.6%), moderate = 2.47, effect higher than 63.7%), large = 4.25, effect higher than 71.6%, and more than large > 8.82, effect higher than 80.2% [46].

## 3. Results

### 3.1. Reliability and Validity of the Protocols

The protocols of the MMPI-A were scrutinized to determine if they had been subjected to extreme acquiescence (TRIN T ≥ 80), random responses (VRIN T ≥ 80; F, F1 or F2 T ≥ 120), lack of collaboration (>10 unresponded or double response items) or outliers (L raw score [rs] > 12 or K rs > 29 i.e., percentile 99.9), in order to eliminate these from the study [47,48]. One case was excluded from the study as lack of collaboration (18 no responses), and another one for random responses (VRIN T > 80).

In the current study, the average ICC for the total score in the PCL-YV was excellent, 0.953 [0.934, 0.966] i.e., 95.3% of the variance and the mean of the raters’ score is true. Thus, data from PCL:YV are reliable.

The reliability for the total score in the YLS/CMI in present study was excellent, average ICC = 0.925 [0.893, 0.947] i.e., 92.5% of the variance and the mean of the scores is true. In consequence, the registered data from YLS/CMI are reliable.

### 3.2. Dangerousness (to Self, Others and/or to the Public)

The results of a MANOVA for the population factor showed non-statistical significant differences in mental health problems, F(8, 125) = 1.76, *p* = 0.091, meanwhile the effect size was between moderate and large (0.59 < η^2^ < 0.137), $\eta_{p}^{2}$ = 0.101, and the observed power was 73.7%, 1 − β = 0.737, i.e., the chance of making a false negative (type II error) was 26.3%. Then, the ratio between type II error and Type I error is 2.89; that is, it is 3.56 times more probable a false negative than a false positive, OR = 3.56. Thus, a significant effect, Z = 14.82, *p* < 0.001, was observed for superiority of type II error over type I error. Inconsequence, although no statistically significant differences were observed between CPOs and non-CPOs offenders in dangerousness, the result is not robust.

However, the probability of clinical deteriorate (prevalence of caseness) among CPOs (see Table 3) was statistically significant when contrasting with clinical significance (i.e., 0.05 corresponding to a T score of 66.45) in hypochondriasis (illness anxiety disorder), depression, hysteria (somatic symptom disorder), psychopathic deviate, paranoia, psychasthenia (generalized anxiety disorder, obsessive-compulsive disorder) and schizophrenia (social alienation in non-clinical populations). Furthermore, the magnitude of the effect for dangerousness (to the public) ranged from a large (>4.25 times more likely clinical deteriorate in CPOs than in normative sample) effect size in hypochondriasis, depression, hysteria, paranoia, psychasthenia and schizophrenia to a more than large (>8.82 times more likely clinical deteriorate in CPOs than in normative sample) effect size in psychopathic deviate. Likewise, 75.0%, 95% CI [0.653, 0.847], of CPOs were caseness; that is, caseness is more than common in CPOs (contrast: 0.50; [45], Z = 4.36, *p* < 0.001, and with a large effect size, h = 0.74, 95% CI [0.64, 0.84], among CPOs), being 18.4% comorbid and 34.2% multimorbid.

### 3.3. Psychopathic Traits

The results exhibited a multivariate significant effect, F(4, 131) = 3.39, *p* = 0.011, whereas the observed power was 84.0%, 1 − β = 0.840, i.e., a 16.0% chance of having a false negative (type II error), accounting for the 9.4% of the variance, $\eta_{p}^{2}$ = 0.094, 95% CI [0.012, 0.156], a moderate (0.059 < η^2^ < 0.137) effect size higher than 67.72% of all possible, PS_ES_ = 0.6772, in psychopathic traits for the population factor. Thus, CPOs and non-CPOs differ in psychopathic traits.

The univariate effects (see Table 4) revealed statistically significant differences in interpersonal and affective psychopathic traits between CPOs, and non-CPOs. Succinctly, CPOs exhibited more interpersonal and affective psychopathic traits with a moderate magnitude effect size. Quantitatively, COPs increases over non-CPOs a 28.7% and a 23.8% in interpersonal and affective traits, respectively. Nevertheless, the error model, i.e., the probability of an effect in CPOs population under the mean of the non-COPS population, was 27.4% and 31.2% for interpersonal and affective traits, correspondingly. 

The observed prevalence of the diagnostic of psychopathy (rs ≥ 30) among CPOs, 25% (n = 19), 95% CI [0.15, 0.35] was statistically significant (contrast: 0.01; estimated prevalence of psychopathy in the general population; [22]), Z = 21.03, *p* < 0.001, and of large effect, h = 1.19, 95% CI [1.09, 1.29].

### 3.4. Risk Factors

The results exhibited a multivariate significant effect, F(8, 127) = 3.34, *p* = 0.002, meanwhile observed power of 96.9%, 1 − β = 0.969, i.e., type II error probability was 3.1%, explaining for the 17.4% of the variance, $\eta_{p}^{2}$ = 0.174, 95% CI [0.029, 0.244], a large (η^2^ > 0.137) effect size higher than 74.22% of all possible, PS_ES_ = 0.7422, in risk factors for the population factor, i.e., CPOs and non-CPOs diverge in risk of recidivism.

The univariate effects (see Table 5) showed significant differences in family circumstances/parenting, and personality and behavior risk factors between CPOs, and non-CPOs. Succinctly, higher scores were observed in CPOs in family circumstances/parenting risk factor of a small to moderate magnitude (0.20 < *d* < 0.50), and in personality and behavior risk factor of a moderate magnitude. Specifically, COPs have a 21.5% and a 27.4% more risk in the family circumstances/parenting and personality and behavior risk factors than non-CPOs. However, the error model was 33.0% and 28.42% for family circumstances/parenting, and personality and behavior.

The observed prevalence (rs ≥ 23) of CPOs classified as high or very (the very high classification category rarely observed in Spanish juvenile offenders; [39]) risk level by criminogenic risk factors was 55.3% (n = 42), 95% CI [0.441, 0.665], a common prevalence (contrast 0.5; [45]), Z = 0.92, *p* = 0.358.

## 4. Discussion

The generalization of the results of the present study are subjected to limitations. First, although clinical protocols were scrutinized for invalid, this is only for extreme responses, in juvenile delinquent protocols overreporting (to avoid treatment obligations) is suspected [49]. Second, actual dangerous persons, as characterized by severe personality disorders, may not respond or respond inconsistently (see dangerousness subsection) to psychometric measures [48]. Third, the inconsistency in the results for dangerousness between the statistical significance (non-significant) and the effect size (between moderate and large), states that Type II error is substantial. Fourth, the errors of the statistical models stablish the limits of the generalization to the populations. Fifth, the raw observed prevalence should not be considered as a benchmark, the true prevalence is a score into the limits of the confidence intervals.

Having in mind the previous limitations to the generalization of the results, the following conclusions are driven. First, dangerousness is not a distinctive characteristic of child-to-parent offenders from other juvenile offenders. Moreover, although the prevalence of severe psychopathology among juvenile offenders is higher than in non-offenders, the relationship between criminal behavior and severe psychopathology is not causal [17]. Causality has been attributed to early traumatic experiences which increase anger, hostility and irritability being symptoms of the neurotic triad disorders in MMPI-A [50]. As for psychotic disorders, positive symptoms (e.g., hallucinations, delusions) are rarely registered before late teens. Contrariwise, negative symptoms (e.g., anhedonia, avolition, asociality) course in adolescence. Thus, subclinical psychotic disorders (negative symptoms) may be diagnosed in adolescence, but psychotic disorders are rarely diagnosed in juvenile offenders [17]. In any case, psychotic disorders may be associated with hostility and aggression, but spontaneous attacks are exceptional (psychopathology causation). Like in mood disorders, aggression in psychotic disorders is consequence of previous experiences of violence, substance abuse and impulsivity [49]. Second, the prevalence of dangerousness (to self, others and/or public) is extraordinarily high among CPOs. As the effective juvenile offender intervention programs, mainly cognitive-behavioral programs, are focused on training offender criminological needs [1,2,3] the intervention is futile for treatment of dangerousness (clinical symptoms). In consequence, socio-cognitive competence interventions should be complemented with clinical treatment for dangerous offenders. Third, psychopathic traits are common (±50%) in CPOs. As psychopathic traits are related to recidivism [51], reluctant to treatment [52] and go jointly [53], intervention with CPOs in psychopathic traits must be broad implying all, i.e., interpersonal, affective, lifestyle and antisocial traits. In this regard, contradictory results about the successful of the treatment (generally measured in recidivism rates) were reported. Thus, [54] found for all treatments studied an average effectiveness of 0.62 (proportion of non-recidivism after treatment) with extraordinary high rates of effectiveness (so high to be the true rates) of 91% for intensive individual psychotherapy, of 82% for eclectic therapy and of 59% for psychoanalytic therapy. Contrariwise, other reviews [55,56] attribute the effectiveness in reducing recidivism to the improvement in psychopaths of the strategies to avoid legal detention as a consequence of the skills acquired in treatment. In any way, psychopathic traits have an indirect effect on emotional intelligence, a critical skill for an effective treatment of psychopathy [57] and the palliation of the emotional clinical symptoms and, by extension, a mitigation of recidivism [58]. Fourth, a superficial, grandiose, and manipulative personality (interpersonal traits) as well as callousness and lack of remorse (affective traits) define CPOs in comparison to non-CPOs. Callousness (the most severe affective trait) do also discriminate of juvenile offenders from non-offenders [59]. In consequence, intervention programs with CPOs should emphasize the focus in the treatment of interpersonal and affective traits. Fifth, the prevalence of high risk of recidivism for CPOs is common, i.e., encompass 50% of the CPOs population. Consequently, secondary intervention programs must be designed for CPOs to mitigate the risk of recidivism. Sixth, CPOs weight higher in family circumstances/parenting, consisting in inadequate supervision, difficulty controlling behavior, inappropriate discipline; inconsistent parenting, father/mother poor relations, and personality and behavior, entailing inflated self-esteem, physically aggressive, tantrums, short attention span, poor frustration tolerance, inadequate guilt feelings, verbally aggressive, criminogenic risk factors than non-CPOs. Hence, family circumstances and parenting which predict higher rates of recidivism for CPOs in comparison to non-CPOs, require additional intervention with CPOs. Seventh, as a consequence of the joining of the elevated prevalence of dangerousness (75.0% of CPOs were caseness, 18.4% comorbid and 34.2% multimorbid), the large (a large effect size over baseline) prevalence observed of COPs diagnosed of psychopathy, and the common prevalence of high-risk cases (50%) according to criminogenic risk factors, the probability of recidivism for CPOs is extreme.

## Figures and Tables

**Table 1:** Distribution of frequencies of disposition orders.

Crime	Frequency (%)
Secured juvenile facility	23 (16.9%)
Weekend arrest	18 (13.2%)
Day center attendant	19 (14.0%)
Ambulatory center treatment	84 (61.8%)
Probation	129 (94.5%)
Community service	6 (4.4%)

Note. Major disposition order; offenders may be sentenced by more than 1 major disposition order.

**Table 2:** Frequencies of crimes of sentenced for non-child-to-parent juvenile offenders.

Crime	Frequency (%)
Crime against property	30 (50%)
Crime against people	29 (48.3%)
Crimes against sexual freedom	8 (13.3%)
Crime against public health (drug trafficking)	3 (5%)

Note. Major categories of crimes and referred to the main charge(s). Offenders may be sentenced by more than 1 main crime.

**Table 3:** Caseness in the MMPI-A basic clinical scales among child-to-parent offenders.

Scale	*f*(*p* [95% CI])	*Z*	*OR* [95% CI]
Hypochondriasis	22 (0.289 [0.187, 0.391])	9.56 ***	7.72 [6.53, 9.14]
Depression	23 (0.303 [0.200, 0.406])	10.12 ***	8.26 [6.98, 9.77]
Hysteria	22 (0.289 [0.187, 0.391])	9.56 ***	7.72 [6.53, 9.14]
Psychopathic deviate	44 (0.579 [0.468, 0.690])	21.16 ***	26.13 [22.09, 30.91]
Paranoia	24 (0.316 [0.211, 0.421])	10.64 ***	8.78 [7.42, 10.38]
Psychasthenia	18 (0.237 [0.141, 0.332])	7.48 ***	5.90 [4.99, 6.98]
Schizophrenia	20 (0.263 [0.164, 0.362])	7.94 ***	6.78 [5.73, 8.02]
Hypomania	4 (0.053 [0.003, 0.103])	0.79	1.06 [0.90, 1.26]

Note. *N* = 76; *f*(*p* [95% CI]): frequency of clinical deteriorate(observed probability) [95% confidence interval]; *Z*: zeta score for the difference between the observed proportion of clinical deteriorate among CPOs and a constant (T score ≥ 66.45); *OR* [95% CI]: odds ratio for the clinical deteriorate [95% confidence interval]; *** *p* < 0.001.

**Table 4:** Univariate effects in psychopathic traits for the population factor (child-to parent offenders vs. non-child-to-parent offenders). Between-subjects effects.

Psychopathic Trait	*F*	*p*	*M*_CPO_	*M*_N-CPO_	*d* [95% CI]	*r*	PIS [95% CI]
Interpersonal traits	12.22	0.001	5.57	4.55	0.60 [0.53, 0.67]	0.287	0.274 [0.199, 0.349]
Affective traits	8.17	0.005	5.45	4.40	0.49 [0.42, 0.56]	0.238	0.312 [0.234, 390]
Lifestyle	0.27	0.606	7.01	6.85	0.09 [0.02, 0.16]	0.045	0.464 [0.380, 0.548]
Antisocial traits	0.50	0.408	5.12	4.85	0.12 [0.05, 0.19]	0.060	0.452 [0.368, 536]

Note. *df*(1, 134). *M*_CPO_: mean of the CPOs; *M*_N-CPO_: mean of the non-CPOs; *d*: *d* [95% CI]: unbiased *d* [95% Confidence Interval]; *r*: incremental in psychopathic trait; PIS [95% CI]: Probability of an Inferiority Score [95% Confidence Interval]; Box’ M = 15.1, *F*(10, 75,833.4) = 1.46, *p* = 0.147.

**Table 5:** Univariate effects in risk of recidivism for the population factor (child-to parent offenders vs. non-child-to-parent offenders). Between-subjects effects.

Risk of Recidivism	*F*	*p*	*M*_CPO_	*M*_N-CPO_	*d* [95% CI]	*r*	PIS [95% CI]
Prior Offenses	1.01	0.417	1.85	2.03	−0.17 [−0.24, −0.10]	−0.085	0.567 [0.484, 0.650]
Family Circumst.	6.90	0.010	4.50	3.92	0.44 [0.37, 0.51]	0.215	0.330 [0.251, 0.409]
Educ/Employment	0.07	0.797	4.33	4.27	0.04 [−0.03, 0.11]	0.020	0.484 [0.400, 0.568]
Peer Relationships	0.09	0.771	1.96	2.02	−0.05 [−0.12, 0.02]	−0.025	0.520 [0.436, 0.604]
Substance abuse	0.21	0.650	2.10	2.22	−0.08 [−0.15, −0.01]	−0.040	0.532 [0.448, 0.616]
Leisure/Recreation	0.37	0.544	1.92	1.82	0.10 [0.03, 0.17]	0.050	0.460 [0.376, 0.544]
Pers./Behavior	11.15	0.001	4.53	3.80	0.57 [0.50, 0.64]	0.274	0.284 [0.208, 0.360]
Attitudes/Orientation	0.72	0.398	2.66	2.88	−0.17 [−0.10, −0.24]	−0.085	0.567 [0.484, 0.650]

Note. *df*(1, 134). *M*_CPO_: mean of the CPOs; *M*_N-CPO_: mean of the non-CPOs; *d* [95% CI]: unbiased *d* [95% Confidence Interval]; *r*: incremental (decremental for negatives) in risk of recidivism; PIS [95% CI]: Probability of an Inferiority Score [95% Confidence Interval]; Box’ M = 23.21, *F*(36, 53,974.9) = 0.60, *p* = 0.971.

## Data Availability

Data are not publicly available due to legal restrictions and access is restricted to court approval.

## References

[1] Bonta J., Andrews D.A. (2024). The Psychology of Criminal Conduct.

[2] Cano-Lozano M.C., Contreras L., Navas-Martínez M.J., León S.P., Rodríguez-Díaz F.J. (2023). Child-to-parent violence offenders (specialists vs. generalists): The role of direct victimization at home. Eur. J. Psychol. Appl. Leg. Context.

[3] Pappas L.N., Dent A.L. (2023). The 40-year debate: A meta-review on what works for juvenile offenders. J. Exp. Criminol..

[4] Buchanan A., Leese M. (2001). Detention of people with dangerous severe personality disorders: A systematic review. Lancet.

[5] Webster C.D., Sepejak D.S., Menzies R.J., Slomen D.J., Jensen F.A.S., Butler B.T. (1984). The reliability and validity of dangerous behavior predictions. Bull. Am. Acad. Psychiatry Law.

[6] Redondo L., Fariña F., Seijo D., Novo M., Arce R. (2019). A meta-analytical review of the responses in the MMPI-2/MMPI-2-RF clinical and restructured scales of parents in child custody dispute. An. Psicol..

[7] Rogers R., Sewell K.W., Martin M.A., Vitacco M.J. (2003). Detection of feigned mental disorders: A meta-analysis of the MMPI-2 and malingering. Assessment.

[8] Sharf A.J., Rogers R., Williams M.M., Henry S.A. (2017). The effectiveness of the MMPI-2-RF in detecting feigned mental disorders and cognitive deficits: A meta-analysis. J. Psychopathol. Behav. Assess..

[9] Goodley G., Pearson D., Morris P. (2022). Predictors of recidivism following release from custody: A meta-analysis. Psychol. Crime Law.

[10] Hanson R.K., Morton-Bourgon K.E., Roesch R., McLachlan K. (2019). The characteristics of persistent sexual offenders: A meta-analysis of recidivism studies. Clinical Forensic Psychology and Law.

[11] van Der Put C.E., Gubbels J., Assink M. (2019). Predicting domestic violence: A meta-analysis on the predictive validity of risk assessment tools. Aggress. Violent Behav..

[12] Aroca Montolío C. (2013). La violencia de hijos adolescentes contra sus progenitores. Reinad.

[13] Cuervo K., Palanques N. (2022). Risk and protective factors in child-to-parent violence: A study of the YLS/CMI in a Spanish juvenile court. J. Child Fam. Stud..

[14] Hoge R.D., Andrews D.A. (2011). Youth Level of Service/Case Management Inventory 2.0 (YLS/CMI 2.0): User’s Manual.

[15] Pusch N., Holtfreter K. (2018). Gender and risk assessment in juvenile offenders: A meta-analysis. Crim. Justice Behav..

[16] Abracen J., Looman J. (2016). Treatment of High-Risk Sexual Offenders: An Integrated Approach.

[17] Grisso T. (2008). Adolescent offenders with mental disorders. Future Child..

[18] Basanta J., Fariña F., Arce R. (2018). Risk-Need-Responsivity model: Contrasting criminogenic and noncriminogenic needs in high and low risk juvenile offenders. Child. Youth Serv. Rev..

[19] Burt G.N., Olver M.E., Wong S.C.P. (2016). Investigating characteristics of the nonrecidivating psychopathic offender. Crim. Justice Behav..

[20] Hawes S.W., Boccaccini M.T., Murrie D.C. (2013). Psychopathy and the combination of psychopathy and sexual deviance as predictors of sexual recidivism: Meta-analytic findings using the Psychopathy Checklist—Revised. Psychol. Assess..

[21] Hare R.D. (1991). The Hare Psychopathy Checklist-Revised (PCL-R).

[22] Hare R.D. (2003). Manual for the Revised Psychopathy Checklist.

[23] Veal R., Ogloff J.R., Marques P.B., Paulino M., Alho L. (2002). The concept of psychopathy and risk assessment: Historical developments, contemporary considerations, and future directions. Psychopathy and Criminal Behavior: Current Trends and Challenges.

[24] Ibabe I., Arnoso A., Elgorriaga E. (2020). Child-to-parent violence as an intervening variable in the relationship between inter-parental violence exposure and dating violence. Int. J. Environ. Res. Public Health.

[25] Cano-Lozano M.C., Navas-Martínez M.J., Contreras L. (2021). Child-to-parent violence during confinement due to COVID-19: Relationship with other forms of family violence and psychosocial stressors in Spanish youth. Sustainability.

[26] Pagani L.S., Tremblay R.E., Nagin D., Zoccolillo M., Vitaro F., McDuff P. (2004). Risk factor models for adolescent verbal and physical aggression toward mothers. Int. J. Behav. Dev..

[27] Ibabe I. (2014). Direct and indirect effects of family violence on child-to-parent violence. Estud. Psicol..

[28] López-Barranco P.J., Jiménez-Ruiz I., Pérez-Martínez M.J., Ruiz-Penin A., Jiménez-Barbero J.A. (2022). Systematic review and meta-analysis of the violence in dating relationships in adolescents and young adults. Rev. Iberoam. Psicol. Salud.

[29] United Nations (1985). Declaration of Basic Principles of Justice for Victims of Crime and Abuse of Power.

[30] Marcos V., Seijo D., Montes Á., Arce R. (2023). Prevalence and quantification of the effects of sexual harassment victimization of school-aged adolescents. Children.

[31] Loinaz I., Villanueva J., Sancho J.L. (2023). Pre-post changes in a child-to-parent violence psychoeducational intervention program. Eur. J. Educ. Psychol..

[32] Maroto Z., Cortés M. Reincidencia y factores psicológicos en jóvenes con conductas de maltrato hacia sus progenitores. [Recidivism and psychological factors in young violence against parents]. Reinad.

[33] Moulds L., Day A., Mayshak R., Mildred H., Miller P. (2019). Adolescent violence towards parents—Prevalence and characteristics using Australian Police Data. J. Criminol..

[34] Butcher J.N., Williams C.L., Graham J.R., Archer R.P., Tellegen A., Ben- Porath Y.S., Kaemmer B. (2003). MMPI-A: Manual.

[35] Ivanova-Serokhvostova A., Molinuevo B., González L., Hilterman E.L.B., Pardo Y., Pera-Guardiola V., Bonillo A., Batalla I., Torrubia R., Forth A. (2023). Psychometric properties of the Spanish version of the Psychopathy Checklist: Youth version. Curr. Psychol..

[36] Forth A.E., Kosson D.S., Hare R.D. (2003). The Psychopathy Checklist: Youth Version.

[37] Nunnally J.C. (1978). Psychometric Theory.

[38] Forth A.E., Mailloux D., Gacono C. (2000). Psychopathy in Youth: What Do We Know?. The Clinical and Forensic Assessment of Psychopathy.

[39] Cuervo K., Villanueva L., Prado-Gascó V. (2017). Predicción de la reincidencia juvenil mediante el inventario YLS/CMI y baremos para su valoración. [Youth recidivism prediction using the YLS/CMI and norms for assessment]. Rev. Mex. Psicol..

[40] Koo T.K., Li M.Y. (2016). A guideline of selecting and reporting intraclass correlation coefficients for reliability research. J. Chiropr. Med..

[41] Cohen J. (1988). Statistical Power Analysis for Behavioral Sciences.

[42] Vilariño M., Amado B.G., Seijo D., Selaya A., Arce R. (2022). Consequences of child maltreatment victimization in internalizing and externalizing mental health problems. Leg. Criminol. Psychol..

[43] Rosenthal R., Rubin D.B. (1982). A simple, general purpose display of magnitude of experimental effect. J. Educ. Psychol..

[44] Arias E., Arce R., Vázquez M.J., Marcos V. (2020). Treatment efficacy on the cognitive competence of convicted intimate partner violence offenders. An. Psicol..

[45] Fandiño R., Basanta J., Sanmarco J., Arce R., Fariña F. (2021). Evaluation of the executive functioning and psychological adjustment of child to parent offenders: Epidemiology and quantification of harm. Front. Psychol..

[46] Arce R., Fariña F., Seijo D., Novo M. (2015). Assessing impression management with the MMPI-2 in child custody litigation. Assessment.

[47] Graham J.R. (2011). MMPI-2: Assessing Personality and Psychopathology.

[48] Greene R.L. (2011). The MMPI-2/MMPI-2-RF: An Interpretive Manual.

[49] American Psychiatric Association (2013). Diagnostic and Statistical Manual of Mental Disorders.

[50] Biederman J., Spencer T. (1999). Depressive disorders in childhood and adolescence: A clinical perspective. J. Child Adolesc. Psychopharmacol..

[51] Salekin R.T. (2008). Psychopathy and recidivism from mid-adolescence to young adulthood: Cumulating legal problems and limiting life opportunities. J. Abnorm. Psychol..

[52] Caldwell M.F., McCormick D., Wolfe J., Umstead D. (2012). Treatment-related changes in psychopathy features and behavior in adolescent offenders. Crim. Justice Behav..

[53] Edens J.F., Marcus D.K., Lilienfeld S.O., Poythress N.G. (2006). Psychopathic, not psychopath: Taxometric evidence for the dimensional structure of psychopathy. J. Abnorm. Psychol..

[54] Salekin R.T. (2002). Psychopathy and therapeutic pessimism: Clinical lore or clinical reality?. Clin. Psychol. Rev..

[55] Harris G.T., Rice M.E., Patrick C. (2006). Treatment of psychopathy: A review of empirical findings. Handbook of Psychopathy.

[56] Reidy D.E., Kearns M.C., DeGue S. (2013). Reducing psychopathic violence: A review of the treatment literature. Aggress. Violent Behav..

[57] Gómez-Leal R., Megías-Robles A., Sánchez-López M.T., Fernández-Berrocal P. (2021). Psychopathic traits and ability emotional intelligence in incarcerated males. Eur. J. Psychol. Appl. Leg. Context.

[58] Molero M.M., Pérez-Fuentes M.C., Martos Á., Pino R.M., Gázquez J.J. (2023). Network analysis of emotional symptoms and their relationship with different types of cybervictimization. Eur. J. Psychol. Appl. Leg. Context.

[59] Padrón I., Góngora D., Moreno I., Rodrigo M.J., Martín A.M. (2022). Contribution of brain cortical features to the psychological risk profile of juvenile offenders. Eur. J. Psychol. Appl. Leg. Context.

